# Prediction and modeling of pre-analytical sampling errors as a strategy to improve plasma NMR metabolomics data

**DOI:** 10.1093/bioinformatics/btx442

**Published:** 2017-07-14

**Authors:** Carl Brunius, Anders Pedersen, Daniel Malmodin, B Göran Karlsson, Lars I Andersson, Gunnel Tybring, Rikard Landberg

**Affiliations:** 1 Department of Biology and Biological Engineering, Chalmers University of Technology, Gothenburg, Sweden; 2 Department of Molecular Sciences, Swedish University of Agricultural Sciences, Uppsala, Sweden; 3 Swedish NMR Centre, University of Gothenburg, Gothenburg, Sweden; 4 Department of Laboratory Medicine, Karolinska Institutet, Stockholm, Sweden; 5 Department of Medical Epidemiology and Biostatistics, Karolinska Institutet, Stockholm, Sweden; 6 Institute of Environmental Medicine, Karolinska Institutet, Stockholm, Sweden

## Abstract

**Motivation:**

Biobanks are important infrastructures for life science research. Optimal sample handling regarding e.g. collection and processing of biological samples is highly complex, with many variables that could alter sample integrity and even more complex when considering multiple study centers or using legacy samples with limited documentation on sample management. Novel means to understand and take into account such variability would enable high-quality research on archived samples.

**Results:**

This study investigated whether pre-analytical sample variability could be predicted and reduced by modeling alterations in the plasma metabolome, measured by NMR, as a function of pre-centrifugation conditions (1–36 h pre-centrifugation delay time at 4 °C and 22 °C) in 16 individuals. Pre-centrifugation temperature and delay times were predicted using random forest modeling and performance was validated on independent samples. Alterations in the metabolome were modeled at each temperature using a cluster-based approach, revealing reproducible effects of delay time on energy metabolism intermediates at both temperatures, but more pronounced at 22 °C. Moreover, pre-centrifugation delay at 4 °C resulted in large, specific variability at 3 h, predominantly of lipids. Pre-analytical sample handling error correction resulted in significant improvement of data quality, particularly at 22 °C. This approach offers the possibility to predict pre-centrifugation delay temperature and time in biobanked samples before use in costly downstream applications. Moreover, the results suggest potential to decrease the impact of undesired, delay-induced variability. However, these findings need to be validated in multiple, large sample sets and with analytical techniques covering a wider range of the metabolome, such as LC-MS.

**Availability and implementation:**

The sampleDrift R package is available at https://gitlab.com/CarlBrunius/sampleDrift.

**Supplementary information:**

Supplementary data are available at *Bioinformatics* online.

## 1 Introduction

Biobanks have become one of the most important infrastructures for research in life sciences and medicine. Modern biobank-based research is not only based on biospecimen, but also integrates medical data and high-throughput comprehensive molecular analysis such as metabolomics. Sample collection and handling, from needle to freezer, is a major logistical challenge and also the largest source of laboratory errors (Anton *et al.*, 2015; Ellervik and Vaught, 2015). Evidence-based practises to minimize pre-analytical errors are critical and have typically been developed based on conventional biochemical assessments, but more research is needed to provide evidence of best practise for -omics techniques, including metabolomics (Yin *et al.*, 2013, 2015). Development and adherence to standard operating procedures (SOPs) can reduce the bias inherent in sample handling, but currently used SOPs are often based on best practises and not on experimental findings (Anton *et al.*, 2015).

Despite large efforts and investments to implement highly standardized procedures and Information and Communications Technology (ICT) systems enabling monitoring and tracking of time and temperatures from needle to freezer in large-scale studies, it may still be difficult to collect and handle samples in an optimal way due to multiple study centers involved and long transportation to central laboratories and freezers (Breier *et al.*, 2014). In several modern large-scale population cohorts, such as UK Biobank, Lifelines in the Netherlands and LifeGene in Sweden, compromises aiming for cost-effectiveness have resulted, for example, in handling flows where samples are kept at 4 °C for up to 24 h until they can be aliquoted and frozen (Almqvist *et al.*, 2011; Anton *et al.*, 2015; Elliott *et al.*, 2008; Jobard *et al.*, 2016; Malm *et al.*, 2016). Such handling conditions have been reported to have limited impacts on many analytes (Elliott *et al.*, 2008; Qundos *et al.*, 2013). However, the metabolome represents great chemical diversity, with plasma stability of individual metabolites spanning a wide range from highly stable to labile, and results from recent metabolomics studies have indeed indicated that changes in the plasma and serum metabolome can occur under conditions typically used in collection and handling of samples for large-scale epidemiological studies (Anton *et al.*, 2015; Bernini *et al.*, 2011; Kamlage *et al.*, 2014; Yang *et al.*, 2013; Yin *et al.*, 2013, 2015). Moreover, it is tempting in many research projects to use large sample collections that have unique metadata associated or contain rare samples from an urgent patient population, but have been stored for decades and typically lack any documentation on how samples have been collected, processed and stored (Moore *et al.*, 2011).

In order to avoid bias in scientific results, researchers need to know whether they can use a specific sample collection to address specific research questions, with the aim of using the right sample collection for the right research question. This calls for development of strategies to avoid such bias. One approach could be to use specific endogenous markers for sample quality, as a way to differentiate samples that have been handled under different conditions (Anton *et al.*, 2015; Trezzi *et al.*, 2016). Another approach could be to model the kinetics of different metabolites in samples over different temperature and time conditions, two important pre-analytical sample management parameters detrimental of quality, and use such models to correct for pre-analytical errors. Predictive modeling could be particularly useful when meta-data on time and temperature are lacking, in order to assess the sample pre-analytical history. In the present study, our aim was to investigate the feasibility of modeling and correcting for effects of pre-centrifugation delay time and temperature on the plasma metabolome, i.e. where handling delay occurs in whole blood, where metabolism is potentially heavily ongoing.

## 2 Materials and methods

### 2.1 Biological samples

For modeling of the effects of pre-analytical sample management on the plasma metabolome, blood samples were collected at Karolinska Institutet Biobank from 16 non-fasting healthy donors, eight males and eight females, in the age range 24–62 years. Blood was drawn into 10 × 4 mL K2-EDTA tubes (BD cat. no. 368861). All samples were gently mixed and stored for 1, 3, 8, 24 and 36 h at 4 °C or 22 °C, before centrifugation at 20°C at 2000 g for 10 minutes, followed by aliquotation into 100 µL fractions in 2D-barcoded micro-tubes (heat-sealed REMP-96-300; Brooks Life Science System) using a Tecan Evo liquid handling robot. All samples were then stored at −80˚C until analysis.

For external validation of modeling performance, we analyzed leftover EDTA plasma from 111 random sample donors collected at various sites as part of different research studies. Sampling conditions were similar to those described above, with the exception that all samples, after gentle mixing at the site of collection were sent to Karolinska Institutet Biobank for centralized processing and storage. The accuracy of the pre-centrifugation time of the external sample set was limited to three levels: 3–8 h, 8–24 h or 24–30 h.

The study was approved by the Regional Ethics Review Board in Stockholm (Dnr 2013/703-32).

### 2.2 NMR analysis

Samples were conditioned at -20 °C over-night before being taken out to thaw at 4 °C. To achieve sufficient sample volume, two aliquots of each sample were pooled in 96-well deepwell plates (Sarstedt cat. No. 82.1971.002) using an Eppendorf Multipette E3. Subsequently, each sample (100 µL) was mixed with buffer (100 µL, 75 mM sodium phosphate pH 7.4, 2 mM imidazole, 0.5 mM 3-(trimethylsilyl)-1-propanesulfonic acid-d_6_, 0.05% sodium azide, 20% v/v D_2_O) in another deepwell plate using a SamplePro L liquid handler (Bruker Biospin, Rheinstetten, Germany). Serum-buffer mix (180 µL) was transferred to 3 mm SampleJet NMR tube racks (Bruker BioSpin, Fällanden, Switzerland) with the SamplePro L. All plates and racks were kept at 2 °C during sample preparation.

NMR data were acquired on an Oxford 800 MHz magnet equipped with a Bruker Avance III HD console, a 3 mm TCI cryo probe, and a cooled SampleJet sample changer keeping sample racks at 6 °C. As part of the pulse calibration, the linewidth of the ^2^H signal was monitored and recursively optimized and evaluated after shimming to allow data acquisition only on samples with a half-height linewidth of 2 Hz or better. A CPMG relaxation filter, perfect echo experiment with excitation sculpting for water suppression was used (‘zgespe’ pulse sequence) for acquisition of 1D data, with a sweep width of 20 ppm, 128 scans, an acquisition time of 2.04 s, a relaxation delay of 1.3 s, and a total CPMG pulse train time of 193 ms. Processing of data entailed 0.3 Hz exponential line-broadening, zero-filling, and referencing to the DSS-d6 peak. Data acquisition and processing were performed with TopSpin 3.2pl6 (Bruker BioSpin, Rheinstetten, Germany). 2D natural abundance ^1^H-^13^C HSQC (‘hsqcetgpsisp2.2’ pulse sequence) and ^1^H-^1^H-TOCSY (‘mlevgpphw5’ pulse sequence) were acquired on the same spectrometer, on 1 h, 4 °C and 36 h, 22 °C pooled samples, in order to aid identification of metabolite signals in the 1D dataset. For the HSQCs, spectral widths of 20 (^1^H) and 100 (^13^C) ppm, 16 scans, 38 ms acquisition time, a 3 s relaxation delay, a ^1^J_C–H_ of 145 Hz and acquisition of 2048 data points (for ^1^H) and 1536 increments (for ^13^C) were used. For the corresponding TOCSY experiments, a spectral width of 13.95 ppm was used in both dimensions, and the other acquisition parameters were 16 scans, an acquisition time of 183 ms, and a relaxation delay of 2 s. A total of 4096 and 1024 points were acquired in the direct and indirect dimensions, respectively. The temperature during all NMR experiments was kept at 25 °C. The 160 samples for modeling and the 111 samples for external validation were analyzed in separate batches, 9 months apart.

### 2.3 Data pretreatment

Processed 1D NMR data were imported into R v 3.2.0 (R Core Team, 2015), aligned using the ‘speaq’ R package v 1.2.1 (Vu *et al.*, 2011), and shift-normalized to TSP at δ = 0. Metabolic features (*n* = 478) were obtained using continuous wavelet transformation peak picking and extracting peak heights at corresponding shifts. Despite the automated shimming and evaluation routine described above, batch effects in line shape were observed between the 160 samples used for modeling and the 111 samples used for external validation, where the latter samples had narrower peak shape and therefore higher peak height/area ratio. To address this batch effect, peak intensities were sample-wise normalized by the probabilistic quotient method (Dieterle *et al.*, 2006). Full R script for feature extraction is available from the authors upon request.

### 2.4 Metabolite identification

A combination of spectral deconvolution with Chenomx 8.2 (Chenomx Inc., Edmonton, Canada), 2D NMR data and the use of the human metabolome database, in combination with an in-house R script for statistical correlation spectroscopy (Cloarec *et al.*, 2005), was used to tentatively assign metabolite signals to specific metabolites.

### 2.5 Predictive modeling of pre-centrifugation temperature

Multivariate predictive modeling of pre-centrifugation temperature using feature data was performed to explore the possibilities to predict pre-centrifugation temperature for the purpose of data correction in such situations where sample management metadata are not available, such as for legacy samples. For the classification between 4 °C and 22 °C, a random forest analysis with unbiased variable selection within a repeated double cross-validation scheme (Supplementary Material) was used (Buck *et al.*, 2016; Filzmoser *et al.*, 2009; Westerhuis *et al.*, 2008). Random forest has previously been observed to produce robust and accurate classification analyses in metabolomics experiments (Gromski *et al.*, 2014; Hochrein *et al.*, 2012; Scott *et al.*, 2013). Model performance was assessed by permutation analysis (*n* = 100) (Szymańska *et al.*, 2012) and externally validated by prediction of pre-centrifugation temperature for 111 samples not included in the model construction. Contributions of variables to modeling results were visualized by a PLS-DA biplot.

### 2.6 Predictive modeling of pre-centrifugation time

Time series data, i.e. from samples with 1, 3, 8, 24 and 36 h delay time before centrifugation, were first separated by temperature into a 4 °C set and a 22 °C set, which were then modeled separately. Predictive modeling was similarly conducted using random forest regression of NMR metabolomics data, using pre-centrifugation time as dependent variable. A similar cross-validation protocol and permutation strategy (*n* = 100) as for the modeling of pre-centrifugation temperature was employed (Supplementary Material). External validation was conducted by prediction of pre-centrifugation time for 111 samples not included in the model construction and known to have been stored at 22 °C during handling before freezing. Contributions of variables to modeling results were visualized by PLS regression biplots.

### 2.7 Modeling of pre-centrifugation time-induced changes

**Fig. 1.**
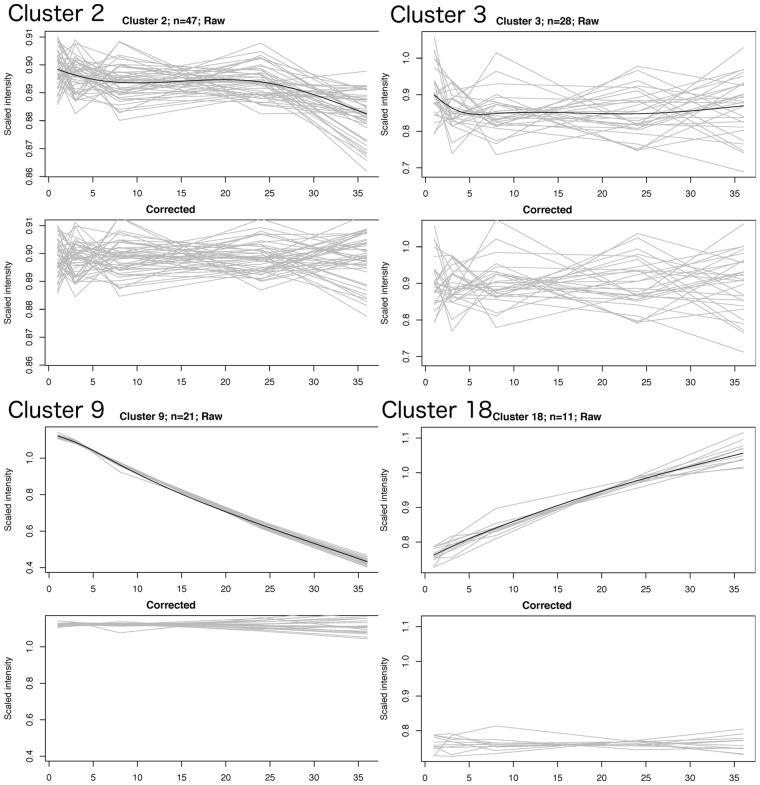
Drift modeling of four clusters with different drift patterns from 22 °C data (Table 1). The two upper graphs represent clusters with small to minimal drift during pre-centrifugation delay time with either significant (left) or non-significant (right) improvement of feature CV after correction. The two lower graphs represent clusters with either decreased (left) or increased (right) feature intensity with increased pre-centrifugation time and significant CV improvement after correction. For each cluster, the upper graph shows the cluster-averaged scaled feature intensities in grey and the cluster drift function in black. The lower half shows the same features in the same y-scale after application of cluster-based drift correction

Modeling was performed separately for 4 °C and 22 °C data. Metabolite features with similar profiles of changes over time were then automatically clustered together (Suppl. Methods), producing *n* = 16 separate drift clusters at 4 °C and *n* = 20 clusters at 22 °C. Cubic spline regression was applied to model the drift profile per cluster (Fig. 1). An analogous approach for data correction was recently developed and successfully applied to correct for instrumental drift in large sample series LC-MS data by clustering and modeling of features with similar instrumental drift patterns (Brunius *et al.*, 2016).

### 2.8 Corrections for errors inherent to pre-centrifugation time

For each sample, change in metabolites over pre-centrifugation time was calculated per metabolite cluster, i.e. for metabolites with similar drift pattern over time, using sample-specific pre-centrifugation time as input, based on either recorded pre-centrifugation time (metadata-approach) or prediction estimates from multivariate modeling (prediction-approach) as time input. The calculated drift was then used to normalize feature data to 1 h pre-centrifugation time (Supplementary Material). Similarly to modeling (above), an analogous approach has recently been successfully developed and applied to correct for instrumental drift in LC-MS data (Brunius *et al.*, 2016).

### 2.9 Software

Modeling of metabolite drift during pre-centrifugation delay and multivariate statistical modeling were performed in the open source statistical environment R v 3.2.0 (R Core Team, 2015). Algorithms, data and workflow are available in the R package ‘sampleDrift’ at https://gitlab.com/CarlBrunius/sampleDrift. Predictive modeling for biomarker discovery of pre-analytical sample management was performed using an in-house R package ‘MUVR’, which is available from the authors on request.

## 3 Results and discussion

Predictive modeling of pre-centrifugation temperature and time was performed to simulate a situation where accurate sample management metadata are not available, e.g. legacy samples or samples collected in-clinic without time-stamping. Applying multivariate predictive modeling, we managed to accurately predict pre-centrifugation temperature and time parameters for later use in data correction.

**Fig. 2.**
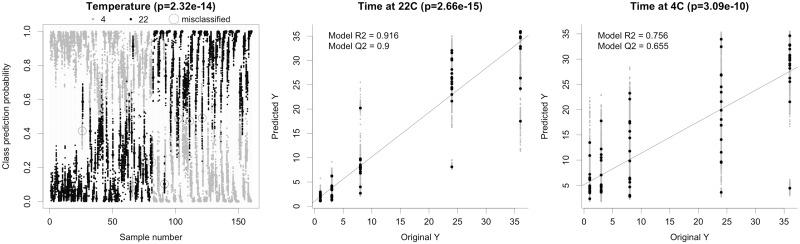
Cross-validated prediction of pre-centrifugation temperature (left) and pre-centrifugation time at 22 °C (center) or 4 °C (right). The pre-centrifugation temperature modeling correctly predicted 96% of samples as either stored at 4 °C (black) or 22 °C (grey). In pre-centrifugation time modeling, predicted times (y-axes) were strongly associated with actual times (x-axes). Predictions were better for pre-centrifugation time modeling at 22 °C, which reflected the larger effects on the metabolome at higher temperatures. All models were highly significant (*P* from permutation analysis; Supplementary Fig. S2)

In the predictive modeling of pre-centrifugation temperature (Fig. 2), only seven of 160 observations (4.4%) were misclassified with respect to pre-centrifugation temperature (p_permutation_=2.32e-14; Supplementary Fig. S2). The results showed that samples undergo reproducible, temperature-dependent alterations in the metabolomic profile within a time frame of approximately 1 h pre-centrifugation time from needle to centrifugation, under standardized and commonly used conditions.

Our results suggest it would be possible, using NMR data only, to differentiate samples lacking accurate metadata on pre-centrifugation temperature, where 4 °C (refrigerator) and 22 °C (room temperature) represent the most likely practical conditions. In total, 14 metabolite features were extracted with the unbiased variable selection procedure as determinants of statistical discrimination between temperatures. These features were primarily identified as pyruvate, lactate and ornithine (Supplementary Table S1; Supplementary Fig. S3), indicating energy and amino acid metabolism as discriminating between pre-centrifugation temperatures. The importance of energy metabolism intermediates as highly discriminating features highlights the absolute need for standardized sampling conditions, in particular the need for an adequate resting period prior to blood withdrawal, since both lactate and pyruvate concentrations in blood are known to be affected by physical exercise (Johnson and Edwards, 1937). The importance of pyruvate as the main driver for temperature prediction was confirmed by comparing the receiver operator curves for the multivariate predictions (Supplementary Fig. S1). Moreover, lactate has previously been shown to increase with time in whole blood, especially at room temperature (Seymour *et al.*, 2011), and was also suggested to be part of a marker of sample quality for metabolomics (Trezzi *et al.*, 2016). The implication of ornithine and possibly arginine supports previous findings by Anton *et al.* (2015), who reported these amino acids to be affected primarily by temperature during delay times. Surprisingly, an imidazole peak at δ = 7.267 was also identified as a discriminating metabolite. Imidazole, which was added in equal amounts to all samples as part of the buffer, had a lower concentration at prolonged delay time, especially at 22 °C (Supplementary Fig. S4) and the high degree of systematic decrease indicated that it was not an artefact. Imidazole was observed to have an irregular peak shape and we thus hypothesized a relationship between imidazole peak height and formation of lactate. However, lactate was found to be negatively correlated also with imidazole peak area (Supplementary Fig. S4). While the reason for the decrease in free imidazole levels is not clear, we speculate that binding with Fe-EDTA complexes (El-Sherif *et al.*, 2012), formed by increased extraction from iron containing proteins on prolonged pre-centrifugation delay may be a possible mechanism, since whole blood total concentration of iron is on the level of 7–10 mmol/L, whereas serum iron concentration is only 10–30 µmol/L. In addition, there may be contributions from photolytic degradation (http://www.inchem.org/documents/sids/sids/288324.pdf) and increased binding with degraded proteins. Other minor, unidentified compounds were also found to discriminate between pre-centrifugation temperatures, although the implications of these findings are not clear.

Modeling of pre-centrifugation time at room temperature resulted in very high prediction accuracy compared with reported pre-centrifugation times (R^2^=0.92, Q^2^=0.90, *P* = 2.66e-15) (Fig. 2; Supplementary Fig. S2). These results clearly show that distinct and reproducible changes in the metabolome occur over time, highlighting the possibility to accurately predict pre-centrifugation time. In the prediction model at 22 °C, 16 metabolite features were extracted as main determinants (Supplementary Table S1; Supplementary Fig. S3). These features belonged predominantly to lactate and glucose, again implicating a considerable and reproducible effect of pre-centrifugation sample management on intermediates of energy metabolism and again highlighting the predictive value of lactate as a marker (Trezzi *et al.*, 2016). Annotation also included imidazole and some unidentified metabolites. Some of the lactate peaks were partially superimposed with threonine, which may possibly strengthen findings by Anton et al. (Anton *et al.*, 2015), who showed that ratios between amino acids, particularly threonine, are indicative of pre-analytical sample management.

**Fig. 3.**
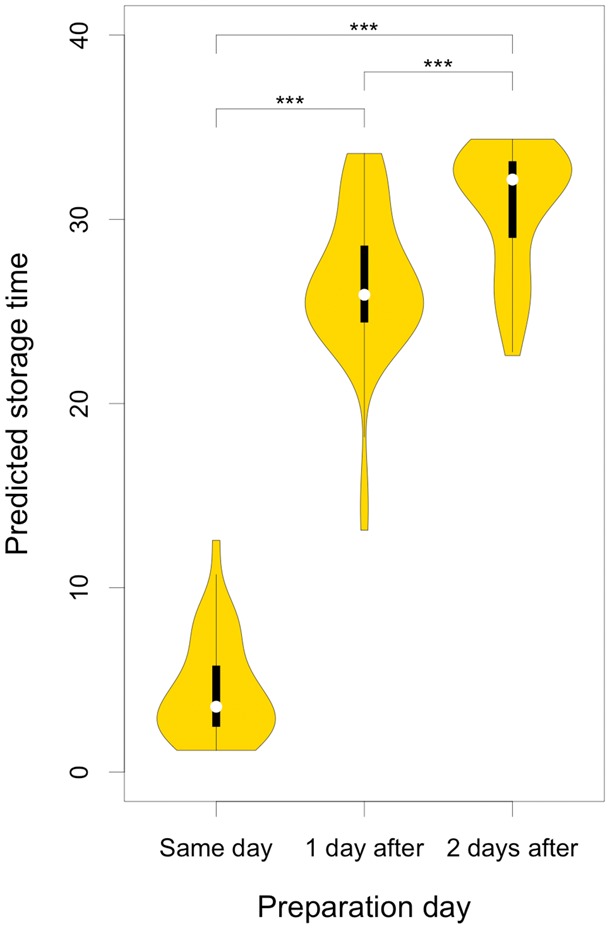
Predicted pre-centrifugation time at 22 °C for external validation samples (*n* = 111). Samples were prepared on either the same day as sampling, the next day, or the day after that. Predicted pre-centrifugation times were significantly different between levels (*P* < 2.2e-16). All pair-wise comparisons were significant after Tukey adjustment (*P* < 0.001)

Predictive performance of modeling at 4 °C was strong, albeit less accurate than modeling at room temperature (R^2^=0.77, Q^2^=0.67, *P* = 3.09e-10) (Fig. 3; Supplementary Fig. S2) and also required more features (*n* = 28) to model metabolite drift inherent to pre-centrifugation time. These features corresponded to lactate, glucose and pyruvate, as well as hypoxanthine, acetate, ornithine, histidine and some other minor and/or unknown compounds (Supplementary Table S1; Supplementary Fig. S3). The reproducible alteration in amino acid profile was in accordance with Anton et al. (Anton *et al.*, 2015), whereas the role of the other, minor compounds is not clear. The consistency with Anton et al regarding the effects of pre-analytical sample management is particularly interesting, considering that their study was performed on serum and handling delay was imposed post-centrifugation, contrary to the present study.

The accuracy of the predictive modeling of pre-centrifugation temperature and time was confirmed by external validation. Only two out of 111 observations were misclassified as being stored at 4 °C. Considering that multivariate modeling was trained on data from only 16 individuals, the results indicate remarkably low inter-person variability in relation to variability induced by difference in temperature, among the selected discriminative features. Unfortunately, exact information about pre-centrifugation times at 22 °C was not available, only information on whether samples were prepared on the same day as blood was drawn, the next day, or the day after that. However, predicted pre-centrifugation times were significantly different between samples prepared on the same day, next day, or day after that (*P* < 2.2e-16, Fig. 3). It should be noted that the predicted pre-centrifugation times were, on average, slightly higher than expected. It is likely that a batch effect between modeling and external validation data could have contributed to this observed discrepancy, since batches were analyzed a substantial time apart. Another potential contributing factor could be that the data used to construct the models were not entirely representative of the external validation dataset, due to e.g. transportation from clinic to central laboratory, seasonal differences, or systematic population differences. Nevertheless, the results in terms of predicted pre-centrifugation times appear very useful for predicting sample metadata.

In the drift modeling, features that showed reproducible drift profiles between individuals were grouped into clusters, representing specific patterns (Tables 1 and 2). This data-mining approach thus resulted in an unbiased representation of multiple drift profiles among the observed features (Fig. 1). At 22 °C, these clusters contained features that had drift that was either low (<10% CV; 29% of features) or moderate (<20% CV; 46% of features), representing metabolites with relative pre-centrifugation stability at 22 °C up to 36 h. There were also clusters containing features with more severe drift (>20% CV; 20% of features), i.e. corresponding to metabolites more severely affected by pre-centrifugation delay time. The clusters that were most affected by pre-centrifugation delay time contained signals predominantly from glucose (decrease), lactate (increase) and pyruvate (increase), as well as several unidentified metabolites, highlighting the effect of pre-centrifugation time on energy metabolism intermediates. Expectedly, pre-centrifugation time at 4 °C increased the proportion of features with a low degree of drift (36% of features) and decreased the proportion with severe drift (13% of features) (Table 2). At 4 °C, the most affected clusters again contained glucose (decrease), lactate (increase) and pyruvate (increase), but also lipids and some amino acids with a specific increase at 3 h, as well as unidentified metabolites (Table 2).

**Table 1.** Cluster-based modeling and data correction for kinetic drift of NMR plasma metabolomics data with pre-centrifugation delay time at 22 °C

		Feature CV^a^			
	*n*	Original	Metadata^b^	Prediction^c^	Dominant identified features
Cluster1	34	0.06	0.05***	0.05***	aas
Cluster2	47	0.058	0.057***	0.058***	3OH-butyrate, citrate, aas
Cluster3	28	0.26	0.26	0.26	Mostly unknown, weak signals
Cluster4	20	0.18	0.16***	0.16***	Alcohols, lipids/ffas
Cluster5	43	0.10	0.086***	0.087***	aas
Cluster6	56	0.056	0.055***	0.055***	aas
Cluster7	20	0.11	0.077***	0.08***	aas, 3OH-butyrate
Cluster8	60	0.11	0.11	0.11	aas, lipids/ffas
Cluster9	21	0.33	0.15***	0.14***	Glucose
Cluster10	34	0.13	0.12***	0.12***	Lipids/ffas, aas
Cluster11	3	0.37	0.33	0.33	Acetate
Cluster12	25	0.11	0.11***	0.11***	Lipids/ffas, 3OH-butyrate, aas
Cluster13	2	0.35	0.35	0.35	Unknown weak signals
Cluster14	14	0.38	0.19***	0.17***	Glucose
Cluster15	12	0.25	0.12***	0.12***	Glucose
Cluster16	2	0.52	0.49	0.46	Glucose (suppressed)
Cluster17	7	0.28	0.1***	0.13***	Pyruvate
Cluster18	11	0.15	0.079***	0.086***	aas
Cluster19	6	0.62	0.11***	0.21***	Lactate
Cluster20	7	0.20	0.14***	0.14***	Lipids/ffas
Modeled	452	0.14	0.10***	0.11***	
Irreproducible	26				Mostly unknown, weak signals
Total	478				

*Note*: Correction for kinetic drift was performed using recorded bench-time metadata or prediction estimates (to simulate legacy samples) as time input. aas, amino acids; ffas, free fatty acids. *p<0.05, **p<0.01, ***p<0.001.

a CV of feature intensities within cluster over 1–36 h.

b After drift correction based on actual pre-centrifugation time from metadata.

c After drift correction based on predicted pre-centrifugation time.

**Table 2.** Cluster-based modeling and data correction for kinetic drift of NMR plasma metabolomics data with pre-centrifugation delay time at 4 °C

		Feature CV^a^			
	*n*	Original	Metadata^b^	Prediction^c^	Dominant identified features
Cluster1	42	0.12	0.12***	0.12**	3OH-butyrate, aas
Cluster2	32	0.17	0.17***	0.17***	aas, alcohols
Cluster3	22	0.30	0.30	0.30	Pyruvate, acetate, histidine
Cluster4	38	0.19	0.19***	0.19***	Sugars
Cluster5	14	0.099	0.097***	0.098*	aas
Cluster6	49	0.094	0.093***	0.094	aas, 3OH-butyrate
Cluster7	36	0.18	0.18***	0.18	Lipids/ffas, 3OH-butyrate, aas, citrate
Cluster8	18	0.35	0.33***	0.34**	Lipids/ffas
Cluster9	13	0.28	0.27***	0.28*	Lipids/ffas, aas
Cluster10	35	0.12	0.097***	0.10***	Glucose
Cluster11	21	0.10	0.10***	0.10***	aas
Cluster12	8	0.10	0.09***	0.091***	Glucose
Cluster13	24	0.085	0.085**	0.085	3OH-butyrate, aas
Cluster14	41	0.075	0.075***	0.075***	aas
Cluster15	7	0.24	0.14***	0.16***	Lactate
Cluster16	44	0.087	0.085***	0.086***	aas
Modeled	444	0.14	0.14***	0.14***	
Irreproducible	34				Mostly lipids/ffas and unknown, weak signals
Total	478				

*Note*: Correction for kinetic drift was performed using recorded bench-time metadata or prediction estimates (to simulate legacy samples) as time input. aas, amino acids; ffas, free fatty acids. *p<0.05, **p<0.01, ***p<0.001.

a CV of feature intensities within cluster over 1–36 h.

b After drift correction based on actual pre-centrifugation time from metadata.

c After drift correction based on predicted pre-centrifugation time.

**Fig. 4.**
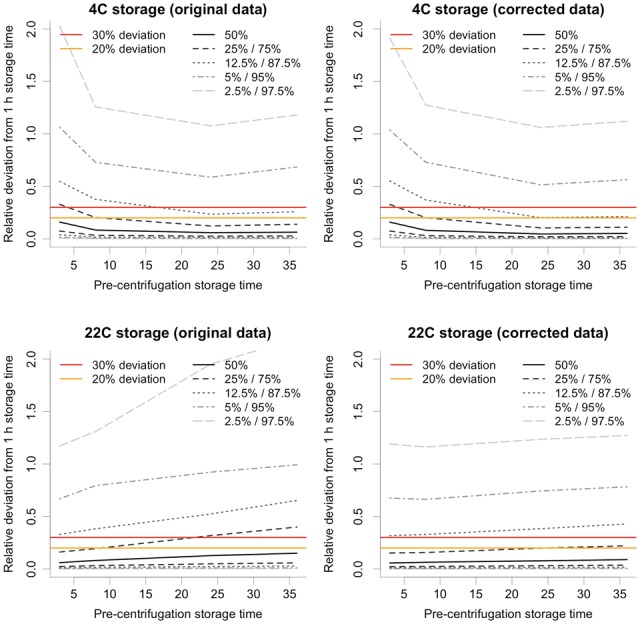
Effects of pre-centrifugation time on metabolite feature stability. The upper graphs show the effects of pre-centrifugation delay time at 4 °C as relative deviation of metabolite feature intensities compared with the reference level (at 1 h) for original data (left) and after data correction based on metadata information (right). Isobaric lines correspond to percentiles in the distribution of relative differences of 7648 measurements (16 samples × 478 features). The lower graphs show corresponding effects of pre-centrifugation delay time at 22 °C. The two horizontal lines correspond to 20% (lower line) and 30% (higher line) absolute deviation in feature intensity from the 1 h reference state

At 22 °C, 26 out of 478 features (5.4%) were considered to have an irreproducible drift pattern due to large inter-individual variability (Supplementary Material), and were therefore removed prior to modeling. Unexpectedly, more features were considered *a priori* irreproducible at 4 °C (34 features; 7.1%) compared with 22 °C. The global variability in feature intensity showed that at 4 °C, the initial pre-centrifugation delay time (especially noticeable at 3 h) contributed to the largest deviations, primarily among lipids, compared with the 1 h reference state (Fig. 4). This increased variability may reflect the comparatively larger temperature gradient from body temperature to refrigerator compared with room temperature, in combination with slow and uneven thermal transfer in the refrigerator. The high irreproducibility at 4 °C could also be due to increased haemolysis upon cooling, with strong inter-individual variation regarding timing and amount of cell rupture. At longer delay times, the number of ruptured cell in individual samples will again become more even, thus decreasing the contribution of haemolysis to irreproducibility. The large observed variability at 4 °C, especially at 3 h refrigerated delay time, deserves special attention in future studies. Global variability in feature intensity at 22 °C, on the other hand, quite expectedly showed variability that increased with pre-centrifugation time and, in general, was higher than at 4 °C (Fig. 4).

Drift was modeled for clusters containing features with specific and reproducible patterns, and the drift patterns obtained were used to correct for the effects of drift, using the pre-centrifugation time from either actual metadata or multivariate prediction (Tables 1 and 2). Both approaches achieved highly significant and approximately equal improvement of the data quality, as measured by a decrease in average feature CV at 1–36 h upon correction both at 22 °C (approx. -26%, *P* < 6.41e-23) and at 4 °C (approx. -3%, *P* < 2.02e-12). Correspondingly, the deviation in feature intensity from reference state at 1 h was decreased (Fig. 4). This was particularly evident at 22 °C, where average feature intensity deviation decreased by up to 60% over the 36 h pre-centrifugation time. A similar, but smaller, decrease of up to 23% was also observed at 4 °C. At 22 °C, there were only four clusters for which there was no significant improvement in data quality (Table 1). These clusters contained either stable lipids and amino acids or low intensity features with high inter-person variability. At 4 °C, only one cluster had no significant improvement in data quality (Table 2; Cluster 3; 22 features). This cluster contained pyruvate, acetate and several unknown, low intensity signals, again with high inter-person variability. Moreover, two clusters had high CV values even after significant improvement (Table 2; Clusters 8 & 9; 31 features), most likely due to a combination of the smaller effect size for correction of 4 °C data and the mentioned irregularities at 3 h delay time. These two clusters contained lipids and amino acids, identification of which suggested N-acetylated amino acids superimposed with proline. The cluster-based drift correction of data at 22 °C reduced the total proportion of features with CV > 30% from a total of 17.2% to 8.2%, including those 5.4% features excluded *a priori* due to irreproducible drift patterns. At 4 °C the corresponding proportion of features with CV > 30% decreased from a total of 15.1% to 14.2%, including those 7.1% features excluded *a priori* due to irreproducible drift patterns. It is apparent that data from samples collected at 22 °C stand to gain more from this data correction approach, although correction at 4 °C is still useful, particularly for those metabolites that appear to be most strongly affected by pre-centrifugation delay times, i.e. energy metabolism intermediates and primarily lactate.

For predictive and drift modeling, data were available only for a limited number of participants. Fully unbiased validation, i.e. complete separation of training, validation and testing sets, was therefore not practically feasible. Statistical overfitting was instead minimized by employing an extended cross-validation framework. Moreover, models for prediction of pre-centrifugation temperature and time were also externally validated using an independent sample set, for which, however, samples were stored only at 22 °C and also with low resolution in pre-centrifugation time metadata. Regardless of these limitations, the external validation showed remarkable potential for prediction of pre-centrifugation sample management history. Still, considering the limited sample size for modeling and also the mentioned limitations in the external validation set, these findings need to be replicated and validated using other, preferably much larger, datasets, both for predictive and drift modeling.

## 4 Conclusions

Predictive modeling effectively distinguished between pre-centrifugation temperatures for samples with ≥1 h pre-centrifugation time, based predominantly on differences in intermediates from energy and amino acid metabolism. At the different temperatures, models were able to accurately predict pre-centrifugation time, especially at 22 °C. These results were confirmed by external validation of 111 EDTA plasma samples. Pre-centrifugation temperature and time had a considerable impact on the metabolite profile and the majority of metabolite alterations occurred in drift patterns that were highly reproducible between individuals. Alterations were more pronounced at 22 °C compared with 4 °C and several distinct drift patterns (clusters), corresponding to specific groups of metabolites, were observed for these alterations. Correction of drift patterns improved the data quality, especially at 22 °C. Pre-centrifugation times up to 36 h at 4 °C had a small effect on the overall metabolite changes captured by NMR, except at 3 h delay time for unknown reasons. Pre-centrifugation delay times at room temperature should be avoided, due to major changes in a large proportion of the metabolites measured. However, the correction approach developed has the potential to diminish the impact of such changes, regardless of whether pre-centrifugation sample management metadata are available, although these findings need to be validated in multiple, large sample sets and with analytical techniques covering a wider range of the metabolome, such as LC-MS. This study improves understanding of the influence of pre-analytical sample handling conditions on metabolomics analysis results and demonstrates the ability of modeling to improve experimental data by taking into account reproducible pre-analytical, sample history-derived changes in individual metabolite levels, where handling conditions (pre-centrifugation temperature and time delay) could be predicted from experimental data.

## Funding

This work was supported by the Swedish Research Council [829-2009-6285] and the European Union Framework Programme 7 [contract 313010] through grants to BBMRI.se and BBMRI-LPC, respectively, and by the Swedish Research Council [2011-2804], the Swedish Research Council Formas [2011-520], the Swedish University of Agricultural Sciences and Chalmers University of Technology through grants to R.L.

*Conflict of Interest*: none declared.

## Supplementary Material

Supplementary Data
