# Supplementary material for: Prediction and modeling of pre-analytical sampling errors as a strategy to improve plasma NMR metabolomics data
Source: Bioinformatics. 2017 Jul 14;33(22):3567–74. doi: 10.1093/bioinformatics/btx442 (PMC5870544; doi:10.1093/bioinformatics/btx442)
Supplement: Supplementary Data [file brunius_et_al_supplementary_2_btx442.docx]

# Supplementary Materials and Methods

## Predictive modeling of pre-centrifugation temperature

To obtain predictive results of classification of pre-centrifugation temperature according to metabolomics profile, a supervised multivariate algorithm was developed using the R software (version 3.2.5). In this algorithm, the ‘randomForest’ function from the ‘randomForest’ package (version 4.6-12) was used as a classifying method within an in-house developed repeated double cross-validation scheme (Filzmoser *et al.*, 2009; Westerhuis *et al.*, 2008) to reduce the probability of statistical overfitting. Furthermore, the random forest was tuned over the number of metabolite features within the inner cross-validation loop to provide an unbiased selection of the most informative features, thereby optimizing model performance (Hanhineva *et al.*, 2015; Buck *et al.*, 2016). In brief, tuning was performed by iteratively performing the inner validation loop using successively fewer features, where in each step of the inner loop the 10% least informative features (decided by average ranking of inner segment models) were removed. The double cross-validation loop was repeated (n=70) to achieve a population of class prediction probabilities per observation, thus enhancing the information content of prediction analysis. Permutation analysis was performed to test overall model validity and degree of overfitting by calculating the cumulative probability of actual model misclassification within a t-distributed H_0_ population. The H_0_ population was in turn populated by number of misclassifications from models (n=100) in which classification labels were randomly drawn without replacement. Student’s t-distribution of H_0_ was assumed and assessed by visual inspection of the H_0_ population histogram (Suppl. Fig. 2).

## Predictive modeling of pre-centrifugation time

Predictive modeling was conducted similarly using random forest regression of NMR metabolomics data, using pre-centrifugation time as dependent variable. A similar cross-validation protocol and permutation strategy (n=100) as for the modeling of pre-centrifugation temperature was employed, but comparing instead model Q^2^ with the Q^2^ distribution of the H_0_ population. Again, Student’s t-distribution of H_0_ was assumed and assessed by visual inspection of the H_0_ population histogram (Suppl. Fig. 2).

## Clustering of metabolite features with similar drift patterns

Time series data were scaled per feature and individual by standard deviation, but not centered. Scaled time series data per feature were then visually examined for reproducibility of feature drift profile between individuals. Drift pattern reproducibility between individuals per feature was numerically assessed by ‘standard distance’ (Eq. 1). Features with an average standard distance >30% were considered not to have sufficiently reproducible drift pattern, and were therefore excluded from further analyses and could thus not be included in any measurement error correction strategy.

| $\forall\left\{ \begin{matrix} i=individual \\ t=time \end{matrix} \right\} standDist=\frac{x_{it}-\bar{x}_{\cdot t}}{\bar{x}_{\cdot\cdot}}$ | Eq. 1 |
| --- | --- |

Many features shared similar drift patterns and features with similar drift patterns were clustered, which improved the drift modeling by providing more data points per model. Clustering of features was performed in the inverse observation/feature space: The underlying assumption being that features with similar drift pattern, in addition to being strongly correlated, are characterized by small Euclidean distances when features are seen as coordinates in the multivariate observation (i.e., pre-centrifugation time) space (Brunius *et al.*, 2016). In brief, clustering of features in the observation space was achieved by the “mclust” algorithm (Fraley and Raftery, 2002; Fraley *et al.*, 2012) (limited to spherical and diagonal models with 1-30 clusters), which employs a Bayesian approach to mine the data for an optimum number of clusters and classification without operator bias. Features were then pooled per cluster.

## Corrections for errors inherent to pre-centrifugation time

For each sample in the data set, drift was calculated per derived metabolite cluster with similar drift pattern, using sample-specific time as input: Drift was calculated using cluster-based cubic spline interpolation models based on either i) the recorded pre-centrifugation time (metadata approach) or ii) prediction estimates from multivariate, random forest modeling (prediction approach) as time input. Drift at that time point was then converted to a scaling factor per drift cluster (Eq. 2), which was used on all features within cluster to normalize data to 1 h pre-centrifugation time. The CV of scaled features for the entire drift profile (1-36 h) was calculated per cluster before and after corrections, as well as one-tailed paired t-tests adjusted by false discovery rate, to assess correction performance.

| $\forall\left\{ \begin{aligned} features within \\ c= cluster \end{aligned} \right\} {scaleFactor}_{c}=\frac{{drift value}_{c, 1h}}{{drift value}_{c, t}}$ | Eq. 2 |
| --- | --- |

## References

Brunius,C. *et al.* (2016) Large-scale untargeted LC-MS metabolomics data correction using between-batch feature alignment and cluster-based within-batch signal intensity drift correction. *Metabolomics*, **12**.

Buck,M. *et al.* (2016) Bacterial associations reveal spatial population dynamics in Anopheles gambiae mosquitoes. *Sci. Rep.*, **6**, 22806.

Filzmoser,P. *et al.* (2009) Repeated double cross validation. *J. Chemom.*, **23**, 160–171.

Fraley,C. *et al.* (2012) mclust Version 4 for R : Normal Mixture Modeling for Model-Based Clustering , Classification , and Density Estimation. *Tech. Rep. 597*.

Fraley,C. and Raftery,A.E. (2002) Model-Based Clustering, Discriminant Analysis, and Density Estimation. *J. Am. Stat. Assoc.*, **97**, 611–631.

Hanhineva,K. *et al.* (2015) Discovery of urinary biomarkers of whole grain rye intake in free-living subjects using nontargeted LC-MS metabolite profiling. *Mol. Nutr. Food Res.*, **59**, 2315–2325.

Westerhuis,J.A. *et al.* (2008) Assessment of PLSDA cross validation. *Metabolomics*, **4**, 81–89.

**Supplementary Table 1**: Explanatory metabolite features for predictive modeling of pre-centrifugation temperature and times at 22°C and 4°C. Metabolite feature lists are listed in order of importance and limited to those variables selected by the respective multivariate models.

| Temperature | |  | Pre-centrifugation time at 22°C | |  | Pre-centrifugation time at 4°C | |
| --- | --- | --- | --- | --- | --- | --- | --- |
| ppm | annotation |  | ppm | annotation |  | ppm | annotation |
| 2.391 | pyruvate |  | 4.122 | lactate |  | 4.122 | lactate |
| 3.075 | ornithine |  | 4.145 | lactate |  | 1.344 | lactate (+ threonine^†^) |
| 7.252 | unknown | | 4.131 | lactate |  | 4.131 | lactate |
| 7.267 | imidazole |  | 4.139 | lactate |  | 1.352 | lactate (+ threonine^†^) |
| 3.084 | ornithine |  | 1.344 | lactate (+ threonine^†^) | | 4.139 | lactate |
| 1.961 | ornithine + arginine | | 1.352 | lactate (+ threonine^†^) | | 4.148 | lactate |
| 1.352 | lactate (+ threonine^†^) | | 4.114 | lactate |  | 4.114 | unknown (lactate) |
| 1.344 | lactate (+ threonine^†^) | | 7.267 | imidazole |  | 8.202 | hypoxanthine |
| 4.114 | lactate |  | 4.229 | unknown |  | 1.938 | acetate |
| 7.800 | unknown |  | 4.220 | unknown |  | 7.815 | histidine |
| 4.122 | lactate |  | 1.464 | unknown |  | 4.062 | unknown |
| 4.131 | lactate |  | 3.477 | glucose |  | 8.220 | hypoxanthine |
| 4.148 | lactate |  | 2.424 | unknown |  | 4.229 | unknown |
| 1.954 | ornithine + arginine | | 3.474 | glucose |  | 7.252 | unknown |
|  |  |  | 3.496 | glucose |  | 7.800 | unknown |
|  |  |  | 1.422 | unknown |  | 3.267 | glucose |
|  |  |  |  |  |  | 3.075 | ornithine |
|  |  |  |  |  |  | 1.961 | ornithine + arginine |
|  |  |  |  |  |  | 7.267 | imidazole |
|  |  |  |  |  |  | 7.694 | unknown |
|  |  |  |  |  |  | 3.084 | ornithine |
|  |  |  |  |  |  | 2.391 | pyruvate |
|  |  |  |  |  |  | 7.075 | unknown |
|  |  |  |  |  |  | 3.915 | glucose |
|  |  |  |  |  |  | 4.208 | unknown |
|  |  |  |  |  |  | 3.874 | glucose |
|  |  |  |  |  |  | 5.762 | unknown |
|  |  |  |  |  |  | 1.954 | ornithine + arginine |

^†^ Threonine is known to be superimposed with lactate at these particular shifts. Νo other threonine peaks were selected in these models, thus indicating lactate as a driver.

**
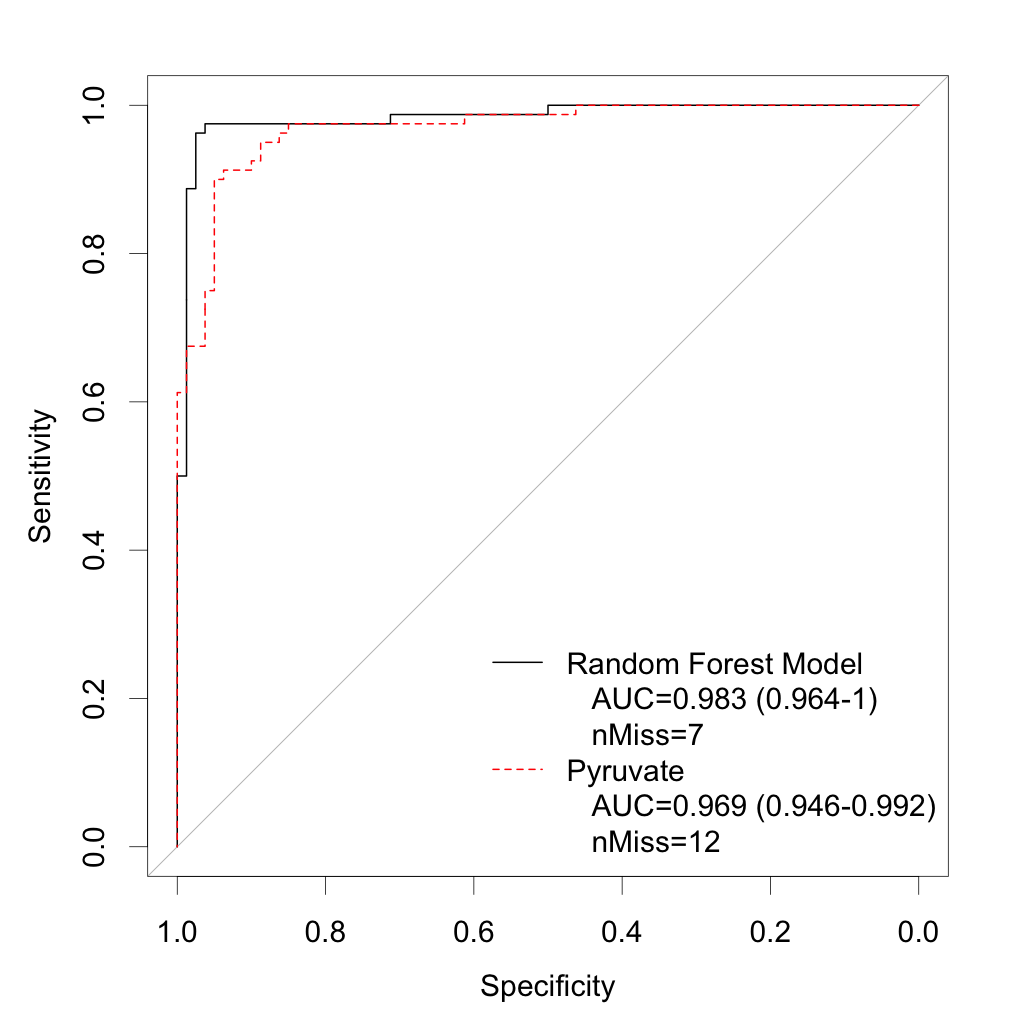
**

**Supplementary Figure 1:** Receiver operator characteristics (ROC) curves for prediction of pre-centrifugation temperature using either multivariate random forest prediction (continuous black line) or pyruvate concentrations (dashed red line).


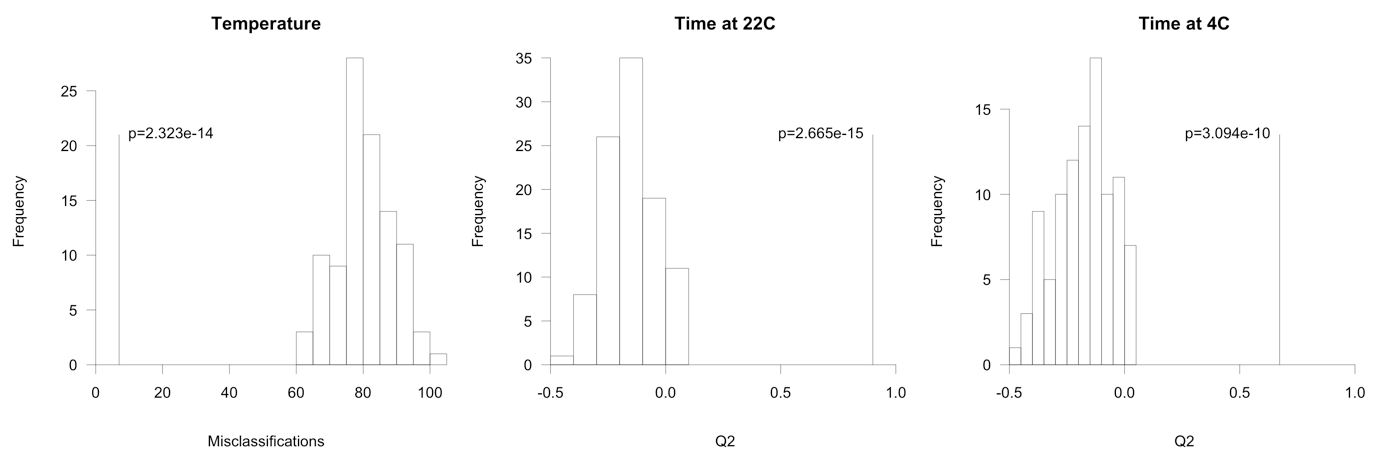


**Supplementary Figure 2**: Permutation analyses (n=100) of multivariate predictive modeling of pre-centrifugation temperature (left), pre-centrifugation time at 22°C (center) and pre-centrifugation time at 4°C (right). Actual model performance is indicated by a vertical line compared with the null hypothesis (permutation) distribution histogram. The close distance between observed and expected H_0_=80 for pre-centrifugation temperature indicates that statistical overfitting in the predictive modeling was negligible. All actual models clearly outperformed random permutations, confirmed by p_permutation_ calculated as cumulative probability in Student’s t-distribution of null hypothesis (permutation) population.


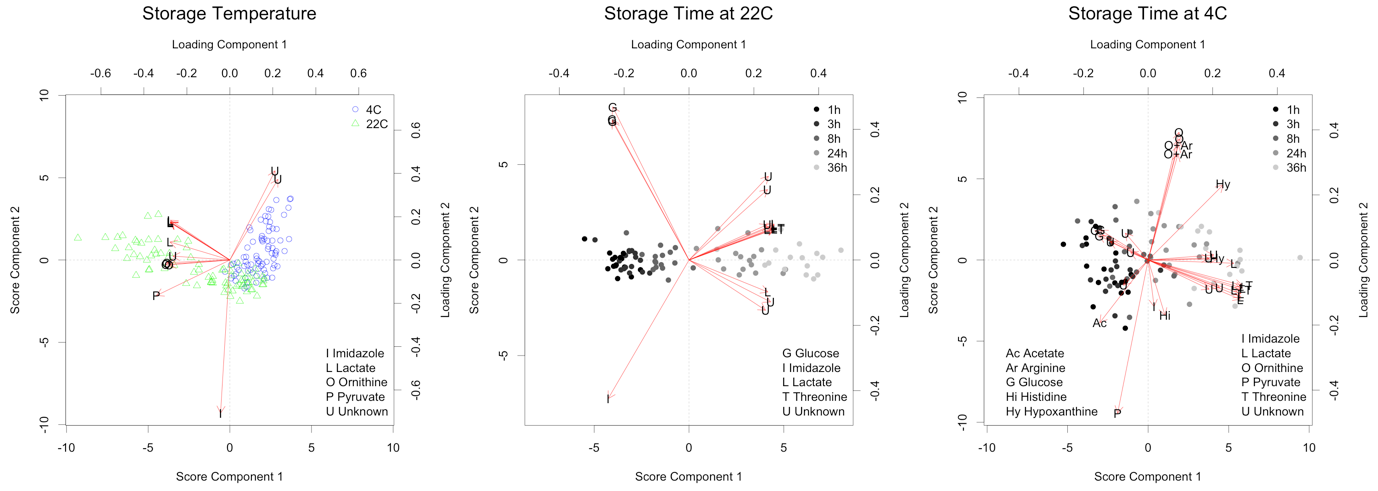


**Supplementary Figure 3**. Visualization of variable contributions to predictive modeling of pre-centrifugation temperature (left) and pre-centrifugation times at 22°C (center) and 4°C (right). Visualizations were achieved as PLS biplots of observation scores and variable loadings (red arrows) using explanatory variables selected from random forest modeling. Observations are coded by subject ID (1-16) and color coded according to legend by either pre-centrifugation temperature (left) or pre-centrifugation delay time (center and right).


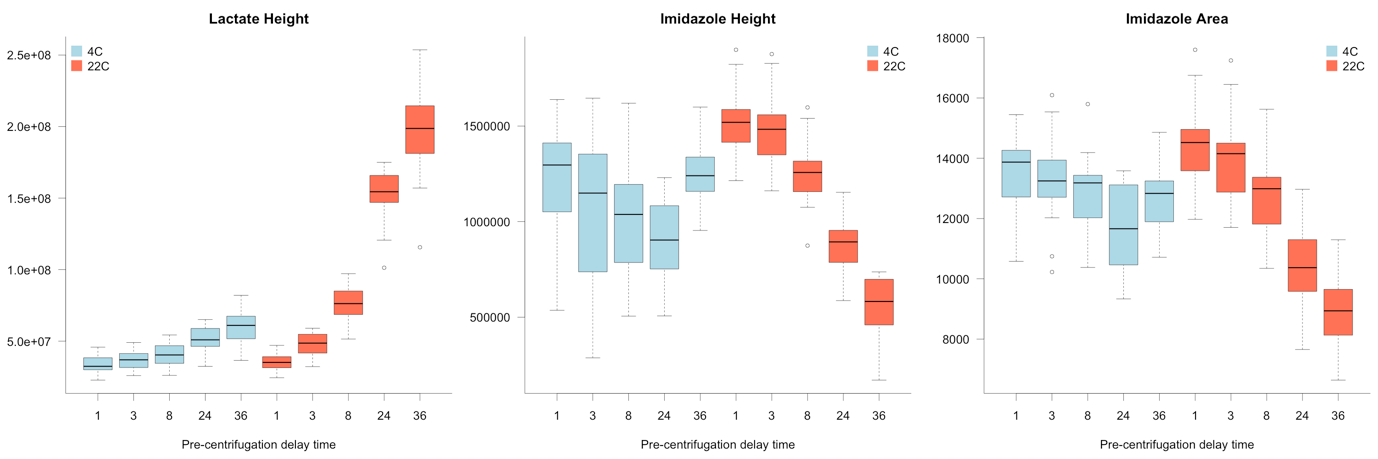


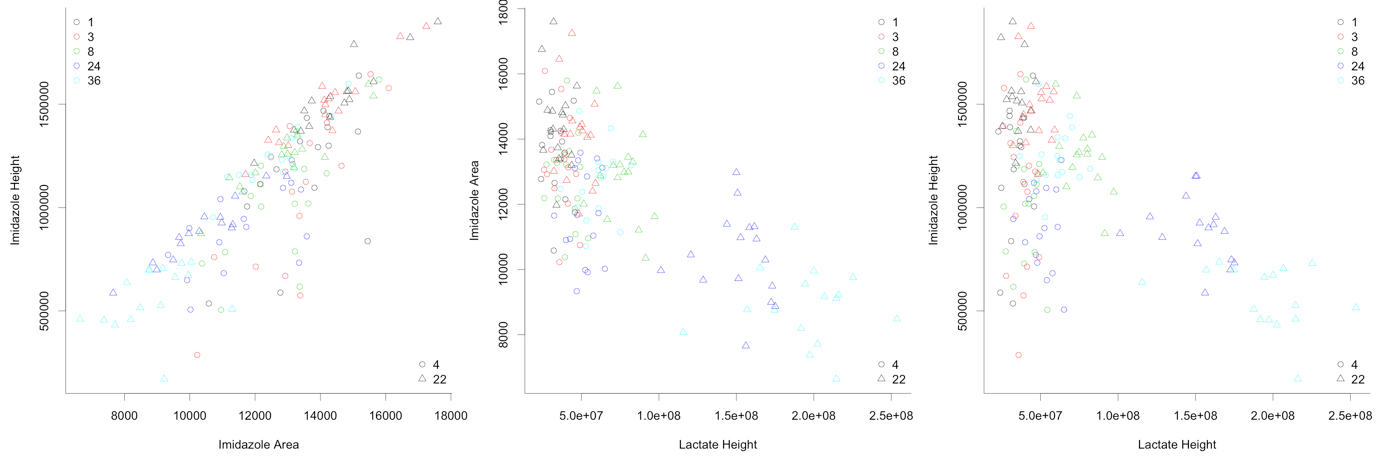


**Supplementary Figure 4.** Upper diagrams: Lactate and imidazole concentrations as a function of pre-centrifugal delay conditions. Lactate levels increased with temperature and delay time, whereas imidazole levels decreased with temperature and delay time. Imidazole was observed to show irregular peak shape. Imidazole levels were therefore investigated by both peak height and peak area. Lower diagrams: Imidazole height and area, showing a positive correlation. Deviations from linearity occurred as a consequence of irregular peak shape. Correlation between imidazole and lactate instead showed a negative correlation, with irregular behavior for imidazole height due to irregular peak shape.
